# Supplementary material for: Angular dependence of the Wigner time delay upon tunnel ionization of H2
Source: Nat Commun. 2021 Mar 16;12:1697. doi: 10.1038/s41467-021-21845-6 (PMC7966791; doi:10.1038/s41467-021-21845-6)
Supplement: Supplementary file 1 — Supplementary Information [file 41467_2021_21845_MOESM1_ESM.pdf]

Supplementary Information

Angular dependence of the Wigner time delay upon tunnel ionization of H_2

D. Trabert[1]*, S. Brennecke[2], K. Fehre[1], N. Anders[1], A. Geyer[1], S. Grundmann[1], M. S. Schöffler[1], L. Ph. H. Schmidt[1], T. Jahnke[1], R. Dörner[1], M. Kunitski[1], and S. Eckart[1]*

[1]Institut für Kernphysik, Goethe-Universität Frankfurt am Main, Max-von-Laue-Straße 1, 60438 Frankfurt am Main, Germany.

[2]Institut für Theoretische Physik, Leibniz Universität Hannover, Appelstr. 2, 30167 Hannover, Germany.

*Corresponding authors: e-mail: (D.T.) trabert@atom.uni-frankfurt.de; (S.E.) eckart@atom.uni-frankfurt.de

Contents

Supplementary Figure 1 | Illustration of the data analysis that is performed to extract $\Delta\alpha$ from the measured electron momentum distributions.

Supplementary Figure 2 | Linking the experimentally accessible quantity $\Delta\alpha$ to changes of the phase gradient of the initial momentum distribution $\Delta\phi'_{\mathrm{init}}$.

Supplementary Figure 3 | Linking the experimentally accessible quantities β and p_{elec} to the values of γ and p_{initial}.

Supplementary Figure 4 | Quantitative presentation of measured and calculated changes of the Wigner time delay.

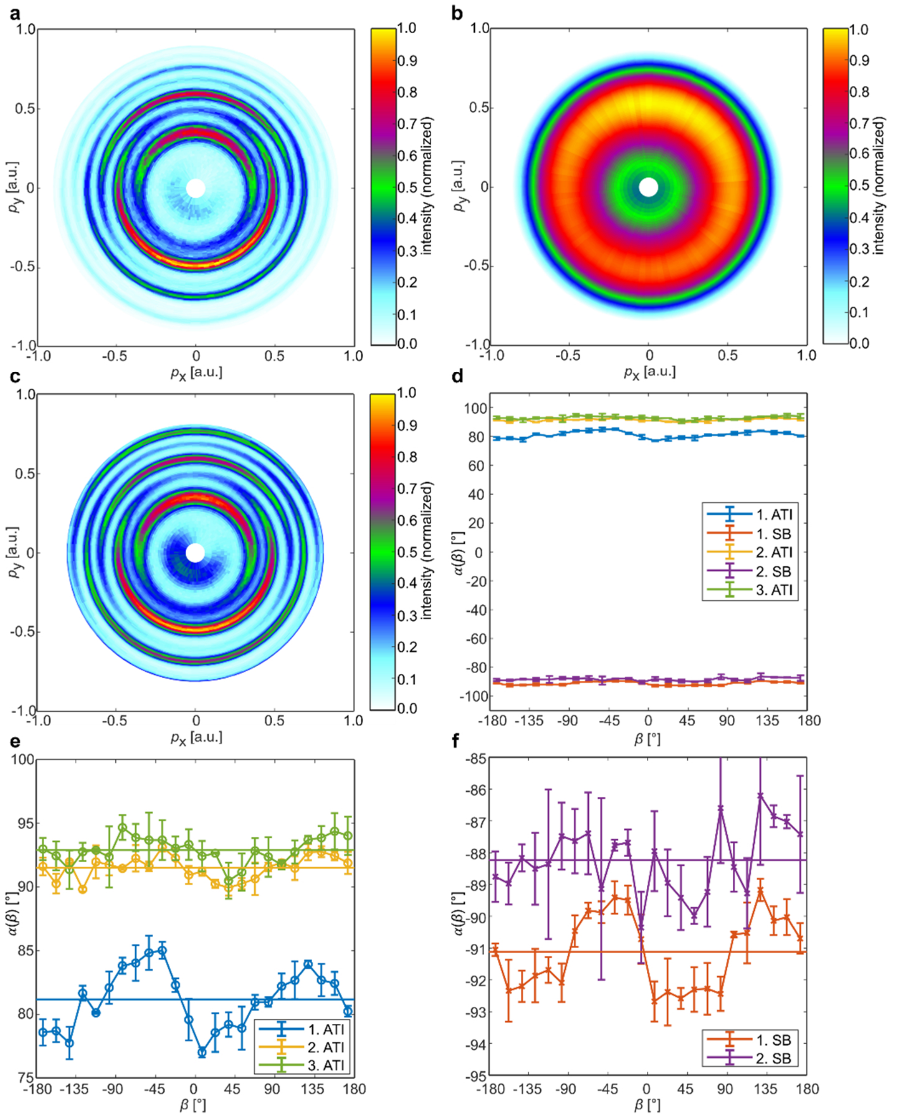

Supplementary Figure 1 | Illustration of the data analysis that is performed to extract $\Delta\alpha$ from the measured electron momentum distributions. a, Measured electron momentum distribution in the polarization plane (only electron momenta corresponding to a kinetic energy $0.1\ eV < E_{\mathrm{kin}} < 11\ eV$ are considered). **b,** The envelope of the electron momentum distribution is retrieved from the full momentum distribution in **a** by filtering higher Fourier components along the radial direction. The obtained envelope is shown in **b**. **c,** Normalized electron momentum distribution obtained by elementwise division of **a** by **b**. This normalization procedure is done for all values of β, that are shown in **d**, separately. **d**, The most probable electron angles, α, are plotted as a function of β (for each energy peak independently). The difference between ATI and SB peaks is approximately 180°. **e, f,** show the same data as **d** but using a different range on the vertical axis. α_{mean} is determined for every energy peak independently as the mean of $\alpha(\beta)$ over all β. The values α_{mean} are depicted as horizontal lines in **e** and **f**. The changes of the most probable electron emission angle as a function of β are defined as $\Delta\alpha(\beta) = \alpha(\beta) - \alpha_{\mathrm{mean}}$. The error bars show the standard deviation of the statistical errors.

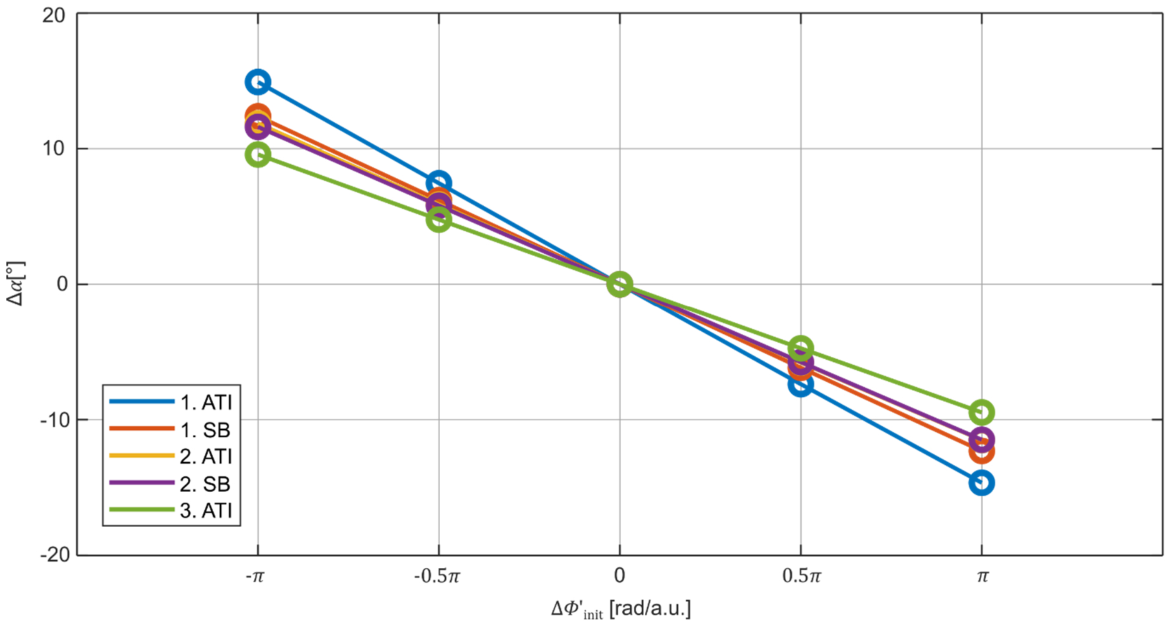

Supplementary Figure 2 | Linking the experimentally accessible quantity $\Delta\alpha$ to changes of the phase gradient of the initial momentum distribution $\Delta\phi'_{\text{init}}$. The result that is shown is obtained from our SCTS calculation (see Methods) and can be read like a look-up-table. This allows for the generation of the values for $\Delta\phi'_{\text{init}}$ in Fig. 3(a) from the data shown in Fig. 2(c). See Fig. S3 for the look-up-table that links the measured values of β and p_{elec} to the values of γ and p_{initial}.

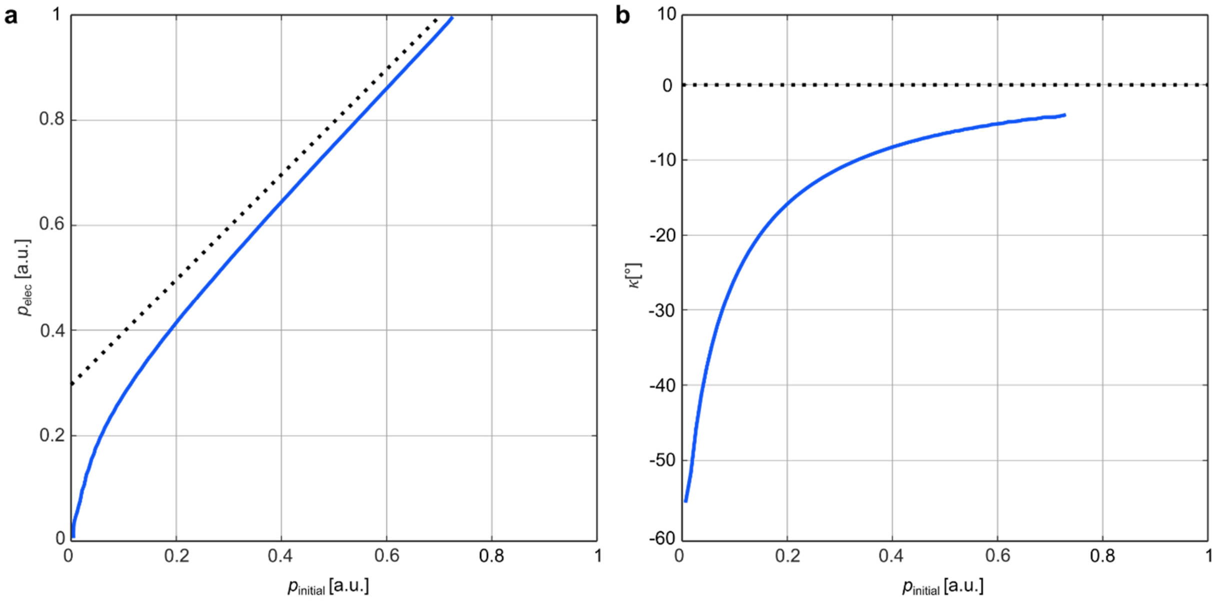

Supplementary Figure 3 | Linking the experimentally accessible quantities β and p_{elec} to the values of γ and $p_{initial}$. a, We calculate the classical trajectories for a circularly polarized light field with a central wavelength of 390 nm and an electric field of 0.037 a.u. and show p_{elec} vs. $p_{initial}$. The blue curve can be read like a look-up-table that is used for the legend of Fig. 3(a). The black dotted line shows the trivial result that would be obtained if Coulomb interaction after tunneling would not be taken into account (the *y*-intercept equals the absolute value of the vector potential A_{390}). **b** can be read like a look-up-table (blue curve) and shows the change in the electron emission direction in the polarization plane that is due to Coulomb interaction after tunneling, which is referred to as κ, as a function of $p_{initial}$. This allows one to infer the value of γ for a given pair of p_{elec} and β using $\gamma = \beta + 90° - \kappa$. The black dotted line shows the trivial result that would be obtained if Coulomb interaction after tunneling would not be taken into account.

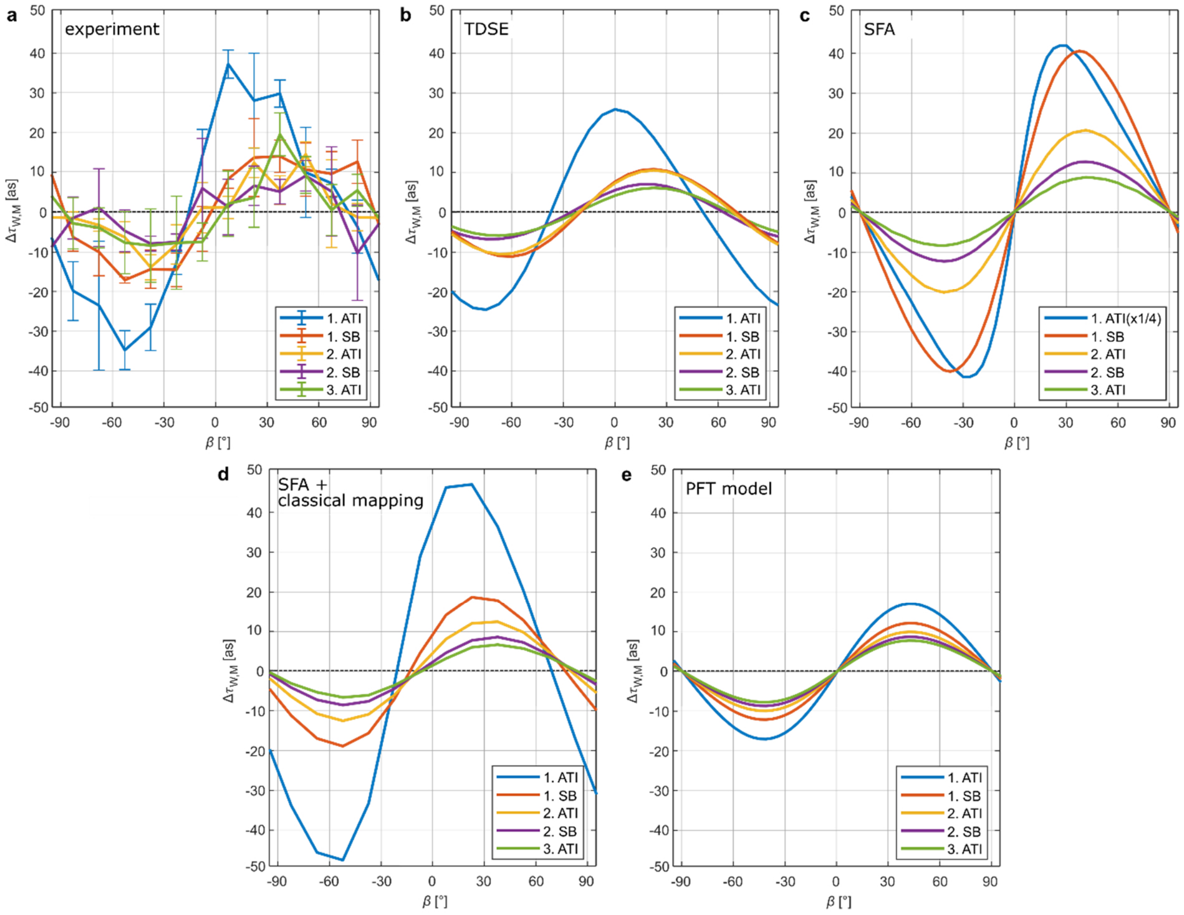

Supplementary Figure 4 | Quantitative presentation of measured and calculated changes of the Wigner time delay. a, Experimentally obtained changes of the Wigner time delay, $\Delta\tau_{\mathrm{W,M}}$ as a function of the electron's emission angle relative to the molecular axis (β). The overall shape of the curves is very similar for the ATI peaks and the SB peaks. The amplitude decreases for higher electron energies. The error bars show the standard deviation of statistical errors (see Methods). **b-e,** show the result from the different theoretical models. Apart from the error bars, **a**, **b, c, d** and **e** show the same data as Fig. 4(a), 4(b), 4(c), 4(d) and 1(d). For the first ATI peak in **c** the values for $\Delta\tau_{\mathrm{W,M}}$ have been scaled as indicated in the legend.
